# Alpha transcranial alternating current stimulation as add-on to neglect training: a randomized trial

**DOI:** 10.1093/braincomms/fcae287

**Published:** 2024-08-30

**Authors:** Marij Middag-van Spanje, Tanja C W Nijboer, Jan Schepers, Caroline van Heugten, Alexander T Sack, Teresa Schuhmann

**Affiliations:** Department of Cognitive Neuroscience, Faculty of Psychology and Neuroscience, Maastricht University, 6200 MD Maastricht, The Netherlands; InteraktContour, 8070 AC Nunspeet, The Netherlands; Department of Experimental Psychology, Helmholtz Institute, Utrecht University, 3584 CS Utrecht, The Netherlands; Center of Excellence for Rehabilitation Medicine, UMC Utrecht Brain Center, University Medical Center Utrecht and De Hoogstraat Rehabilitation, 3583 TM Utrecht, The Netherlands; Department of Methodology and Statistics, Faculty of Psychology and Neuroscience, Maastricht University, 6200 MD Maastricht, The Netherlands; Limburg Brain Injury Center, Maastricht University, 6200 MD Maastricht, The Netherlands; Department of Neuropsychology and Psychopharmacology, Faculty of Psychology and Neuroscience, Maastricht University, 6200 MD Maastricht, The Netherlands; Department of Cognitive Neuroscience, Faculty of Psychology and Neuroscience, Maastricht University, 6200 MD Maastricht, The Netherlands; Maastricht Brain Imaging Centre (MBIC), Department of Cognitive Neuroscience, Faculty of Psychology and Neuroscience, Maastricht University, 6200 MD Maastricht, The Netherlands; Centre for Integrative Neuroscience, Faculty of Psychology and Neuroscience, Faculty of Health, Medicine and Life Sciences, Maastricht University, 6200 MD Maastricht, The Netherlands; Department of Cognitive Neuroscience, Faculty of Psychology and Neuroscience, Maastricht University, 6200 MD Maastricht, The Netherlands; Maastricht Brain Imaging Centre (MBIC), Department of Cognitive Neuroscience, Faculty of Psychology and Neuroscience, Maastricht University, 6200 MD Maastricht, The Netherlands

**Keywords:** stroke, neglect, transcranial electrical stimulation (tES), randomized controlled trial (RCT), activities of daily living (ADL)

## Abstract

Visuospatial neglect is a common and debilitating condition following unilateral stroke, significantly impacting cognitive functioning and daily life. There is an urgent need for effective treatments that can provide clinically relevant and sustained benefits. In addition to traditional stroke treatment, non-invasive brain stimulation, such as transcranial alternating current stimulation, shows promise as a complementary approach to enhance stroke recovery. In the current study, we aimed to evaluate the additive effects of multi-session transcranial alternating current stimulation at alpha frequency when combined with visual scanning training in chronic stroke patients with visuospatial neglect. In this double-blind randomized controlled trial, we compared the effects of active transcranial alternating current stimulation at alpha frequency to sham (placebo) transcranial alternating current stimulation, both combined with visual scanning training. Both groups received eighteen 40-minute training sessions over a 6-week period. A total of 22 chronic visuospatial neglect patients participated in the study (active group *n* = 12, sham group *n* = 10). The median age was 61.0 years, with a median time since stroke of 36.1 months. We assessed the patients at six time-points: at baseline, after the first, ninth and eighteenth training sessions, as well as 1 week and 3 months following the completion of the combined neuromodulation intervention. The primary outcome measure was the change in performance on a visual search task, specifically the star cancellation task. Secondary outcomes included performance on a visual detection task, two line bisection tasks and three tasks evaluating visuospatial neglect in daily living. We found significantly improved visual search (primary outcome) and visual detection performance in the neglected side in the active transcranial alternating current stimulation group, compared to the sham transcranial alternating current stimulation group. We did not observe stimulation effects on line bisection performance nor in daily living. Time effects were observed on all but one outcome measures. Multi-session transcranial alternating current stimulation combined with visual scanning training may be a more effective treatment for chronic visuospatial neglect than visual scanning training alone. These findings provide valuable insights into novel strategies for stroke recovery, even long after the injury, with the aim of enhancing cognitive rehabilitation outcomes and improving the overall quality of life for individuals affected by this condition.

**Trial registration**: ClinicalTrials.gov; registration number: NCT05466487; https://clinicaltrials.gov/ct2/show/NCT05466487

## Teaser

Non-invasive brain stimulation (NIBS) offers the potential to facilitate stroke recovery. The study by Middag-van Spanje *et al*. applied electrical stimulation to chronic stroke patients suffering from visuospatial neglect during 6 weeks rehabilitation. NIBS significantly improved visual search and detection, suggesting a potential treatment option.

## Graphical Abstract

**Graphical abstract:**
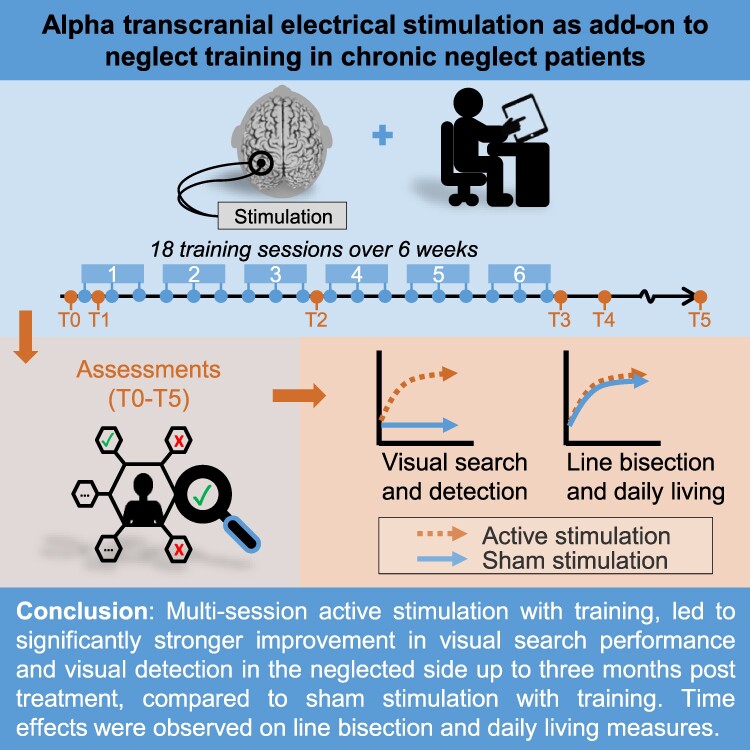

## Introduction

Visuospatial attention allows us to select and prioritize input from specific locations in space of our visual environment. In patients with visuospatial neglect (VSN), lateralized spatial attention processes are disrupted, usually due to unilateral stroke, leading to the inability to attend and respond to the contralesional side of space.^1,2^ While spontaneous neuronal recovery occurs in many VSN patients,^3^ up to 40% of patients continue to experience neglect symptoms even up to 1 year post-stroke.^4^ VSN is a strong predictor of poor functional recovery and significantly impairs activities of daily living (ADL).^5-8^ As such, adequate treatment of neglect is of utmost importance.

In recent decades, a wide range of rehabilitation methods has emerged to attenuate neglect symptoms, spanning from those that enhance awareness of neglect behavior through a top–down approach to those involving a low-level bottom–up approach with many trials and few therapeutic-guided cueing.^9^ Current neglect treatment guidelines primarily recommend behavioral compensation-based approaches such as visual scanning training (VST),^10^ aimed at improving viewing and searching behavior through top–down strategies^11^; yet, the supporting evidence for these methods remains limited.^9,12,13^ However, it should be noted that the review by Longley *et al*. ignores crossover design studies thereby not giving a full picture of all existing studies.^12^ In past years, non-invasive brain stimulation (NIBS) techniques have been explored as a potential rehabilitation tool aimed at directly modulating brain network activity implicated in visuospatial processing. NIBS techniques in rehabilitation treatment are often based on the interhemispheric rivalry model proposed by Kinsbourne in 1977,^14^ often utilizing inhibitory stimulation protocols to reduce contralesional cortical excitability and restore interhemispheric balance in VSN patients. Yet, while promising, reported clinical effects of these NIBS interventions for VSN have remained small and heterogeneous.^12,15,16^

Recently, researchers have focused on NIBS techniques that utilize oscillatory-based neural entrainment, capable of modulating the intrinsic brain rhythms associated with brain network communication. Transcranial alternating current stimulation (tACS) has gained attention for its ability to entrain or synchronize neural oscillations. This entrainment significantly enhances the coherence and power of these oscillations, thereby influencing associated network communications, cognitive processes and behavior.^17,18^

To comprehend the association between oscillatory frequencies and cognitive processes, neuroimaging techniques such as electroencephalography (EEG) have proven invaluable. In the realm of attention, EEG studies in healthy participants have shown that posterior oscillatory activity within the alpha range (8–12 Hz) is crucially involved in the mechanisms underlying the control of visuospatial attention.^19-24^ Voluntary shifts of attention towards one visual field are associated with oscillatory alpha lateralization in parieto-occipital areas. For example, shifting attention to the right hemifield is accompanied by alpha power decreases in the left hemisphere and alpha power increases in the right hemisphere. The successful tACS-induced modulation of alpha power lateralization in healthy individuals including corresponding improvements in visuospatial attention,^24-27^ indicate that such an entrainment-based neuromodulation approach may also represent a novel treatment approach for patients suffering from asymmetric attentional deficits like VSN. It must be mentioned here that several other previous experiments have reported no or inconsistent effects.^28-30^

We recently put this oscillation-based NIBS intervention to the test in subacute stroke patients suffering from VSN, and were able to reduce the spatial attention bias with tACS at alpha frequency targeting the contralesional posterior parietal cortex in a single session.^31^ Besides immediate stimulation effects, we were also able to show that effects were outlasting the stimulation itself, suggesting that our approach qualifies for a clinical treatment protocol aimed at achieving longer lasting and sustainable clinically relevant improvements. It is likely that long-term benefits would require a multi-session multi-day protocol, like is demonstrated in depression treatment with rTMS,^32^ but to this day the cumulative effects of multi-session tACS remain largely unknown as extended human trials with tACS are lacking.^17^

Importantly, when designing clinical protocols using neuromodulation techniques, one should consider that one of the most compelling aspects of tACS is its capacity to support neuroplasticity. As such, it induces a brain state at which the effects of other treatments are facilitated, potentially amplifying both the magnitude and duration of its benefits.^17^ The impact of tACS on the local neural entrainment is contingent upon the state of the brain,^33,34^ as brain networks tend to be more responsive when they are already in an active state. For example, when the targeted brain rhythm is already task-engaged and the frequency and phase of endogenous and exogenous oscillations align.^17,35^ Thus, neuronal plasticity induced by stimulation could be stronger when patients are currently active in a spatial training task. Therefore, to prime the brain for optimal learning conditions and to optimize the outcomes of the treatment, it is important that required attentional networks are activated through attention task performance (VST), executed concurrently with the application of tACS.

In the current study, we therefore combined tACS at alpha frequency with VST rehabilitation in a multi-session protocol, offered three times a week for 6 weeks (18 sessions). The overall aim was to evaluate the additive effects of multi-session (active) tACS in combination with VST, compared to multi-session sham (placebo) tACS with VST, in chronic VSN patients. Effects were measured on a cancellation task (primary outcome), a visual detection task, two line bisection tasks and on three measures assessing VSN behavior in basic ADL. Based on our previous work, we expected to achieve a synergistic effect in which tACS strengthens the efficacy of other neurobehavioral interventions, such as VST, and potentially lead to long-lasting benefits.^17^

## Materials and methods

### Study design

A double-blind randomized placebo-controlled intervention study with an allocation ratio of 1:1 was conducted. We compared the effects of active tACS to sham tACS, both combined with VST. Written informed consent was obtained from each participant by the researcher before participation. The study was approved by the Medical-Ethical Committee azM/UM of Maastricht University (NL70256.068.19/METC 19-047) and is registered at ClinicalTrials.gov (NCT05466487). More specific information of the methodology of the study can be obtained from our protocol paper published earlier.^36^ At the time of registration (July 2022), 13 out of 22 patients had already participated in the study.

### Participants

Chronic stroke patients, as defined by stroke occurrence more than 6 months ago, with VSN were considered eligible for our study. Patients were recruited by psychologists of healthcare organizations in The Netherlands that are specialized in supporting and treating people with acquired brain injury. Recruitment of participants started in September 2020 and data collection ended in March 2023.

Inclusion criteria were: (i) neurologically objectified stroke (first or recurrent, ischaemic or intracerebral or subarachnoid haemorrhagic lesion); (ii) stroke occurred at 18–80 years of age; (iii) at least 6 months ago; (iv) sufficient ability to comprehend and communicate as assessed by a psychologist; and (v) presence of VSN as confirmed with a screening (see screening tests and associated cut-off criteria in Supplementary Table 1). Exclusion criteria were: (i) current engagement in cognitive rehabilitation treatment or other neglect treatment to avoid potential cross-contamination; (ii) physically or mentally unable to participate as assessed by a psychologist; (iii) presence of hemianopia based on clinical judgement; (iv) severe communicative disability as task descriptions need to be understood; (v) local scalp injuries; (vi) eczema on scalp or psoriasis; (vii) diagnosed (neuro)psychiatric or neurodegenerative diseases; (viii) current alcohol and/or drug abuse; and (ix) pregnancy. Excluding patients with hemianopia means that it is possible that there was a stronger focus on more restricted middle cerebral artery strokes, primarily located outside the temporal lobe (due to the presence of the optic radiations there^37^) and the inferior parts of the parietal lobe.

We determined the required sample size based on the results of previous studies that combined VSN treatment with NIBS in repeated sessions.^38-41^ Following an a priori power analysis with effect size of *d* = 0.80 and power of 0.80, and taking into consideration a drop-out estimated at 25%, a total sample size of 22 patients was necessary.^36^

### Interventions

#### Transcranial alternating current stimulation

**Figure 1:**
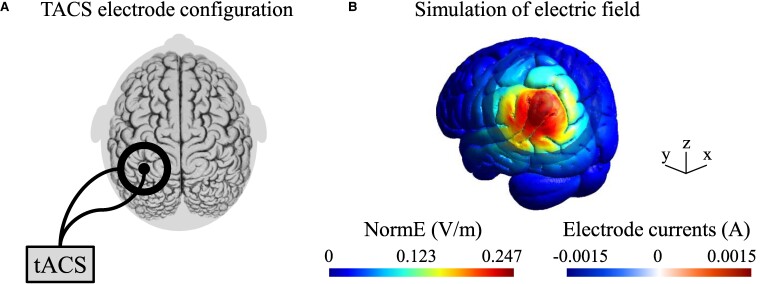
**TACS electrode montage and simulation of the electric field. (A)** A small circular tACS electrode (diameter: 2.1 cm, thickness: 2 mm) was placed onto the contralesional parietal cortex (either P3 or P4, according to the international 10–20 system) and a large ring electrode (outer diameter: 11 cm; inner diameter: 9 cm, thickness: 2 mm) was centred around it. **(B)** Simulation of the electric field in a standard head model (MNI 152 space). The software SimNIBS was used to run the simulation. Abbreviations: A, ampere; normE, norm electric field; tACS, transcranial alternating current stimulation; V/m, volt per metre.

The experimental group received active tACS and the placebo group received sham tACS using a DC-stimulator plus (NeuroConn, Ilmenau, Germany). A small circular tACS electrode was placed onto the contralesional parietal cortex (either P3 or P4, according to the international 10–20 system), and a large ring electrode was centred around it. TACS ring electrodes were attached to the patient’s head with conductive gel (ten20 paste, Weaver and Company, Aurora, CO, USA). The gel was used to reduce the impedance between skin and electrodes to below 10 kΩ. Stimulation frequency and peak-to-peak intensity was set to 10 Hz and 1.5 mA, phase offset was set to zero and 100 cycles was used for ramping up. Figure 1 illustrates the size and position of the electrodes on the scalp as well as a current simulation for the electrode montage.

At the start of every first VST task in a session, the tACS device was turned on. When the VST ended, after maximally 40 minutes, the tACS was switched off. For sham tACS, we used the same electrode montage and stimulation frequency as for active tACS, but the current was immediately ramped down after the ramp up period.

#### Visual scanning training

In every session, patients performed the VST on a touchscreen laptop (HP EliteBook × 360 1040 G5 Notebook; screen size: 14 inch). The aim of the VST was to train patients to actively explore and consciously pay attention to stimuli on the contralesional side.^10,36^ The VST program comprised eight evidence-based training tasks.^36^ In these tasks, while predominantly employing top–down techniques relying on a voluntary effort from the patient, bottom–up elements such as exogenous cues were also integrated if the patient showed difficulty in initiating head and eye movements. Each session featured a variable combination of three to five tasks, depending on the patient’s speed and performance. Tasks were designed with multiple levels of difficulty, ensuring task difficulty aligned with the patient's level of performance, as individuals varied substantially in their capabilities and neglect severity. This allowed for individualized sessions, with different tasks to be conducted in each session, ultimately ensuring that all tasks were covered over multiple sessions.

### Primary outcome measure: star cancellation task (SCT)

The SCT consisted of 52 large stars, 13 letters and 10 short words interspersed with 56 smaller stars,^42^ presented on a laptop screen. The patient was instructed to mark all targets (small stars) by touching the screen with the finger. The quality of search (QoS) score combines accuracy and speed in a single measure (i.e. optimal accuracy/speed search ratio; see formula in Supplementary Table 2).^43^ A high score reflects a combination of a high number of crossed out targets and a high cancellation speed.^43^

### Secondary outcome measures

#### Computerized visual detection task (CVDT)

The CVDT measures perceptual sensitivity and attentional selection in each hemifield.^24,31,44,45^ The patient was asked to fixate on the fixation cross at the centre of the laptop screen. Gabor patches were presented to the left, right and bilateral sides of the screen (40 trials per location) and the patient had to indicate the location of the stimulus by pressing the <, > or ∨ key, respectively. For each of the three locations independently, the contrast of the stimuli was adaptively changed on a trial-by-trial basis. For (offline) analysis, correct hits were weighted by the contrast level (see formula in Supplementary Table 2) and performance of the CVDT was the sum of weighted hits per condition (ipsilesional/contralesional/bilateral), resulting in a score of 0 to 76.49 per condition. As we expected attention deficits in the contralesional hemifield,^31^ our primary focus was directed towards the analyses of the contralesional and bilateral conditions and we only briefly reported on the ipsilesional condition. In the bilateral condition, the score depends on performance in both contralesional and ipsilesional hemifields.

#### McIntosh line bisection task-digitized (MLBT-d)

The MLBT-d was used to measure the so-called endpoint weightings bias (EWB), a measure of lateral asymmetry.^46,47^ There were eight repetitions of each of four unique lines, presented in a fixed-random order on the laptop screen. The patient was instructed to mark the subjective midpoint of each line by touching the screen with the finger. The analysis then focuses on how this response position varies from trial-to-trial as a consequence of changes in the left endpoint and changes in the right endpoint (see formulas in Supplementary Table 2). The EWB value above zero indicates a greater influence of the right endpoint (over the left) and would be a sign for left-sided neglect.

#### Schenkenberg line bisection task (SLBT)

The SLBT consisted of 20 horizontal lines, varying from 10 to 20 cm in length, at three different positions (left, middle and right) on a landscape-oriented A4 sheet.^48^ The patient was asked to mark their perceived midpoint of each line. The relative deviation scores were then calculated (see formula in Supplementary Table 2) and were averaged per line position to generate the left, middle and right average scores. We analyzed only the lines positioned on the contralesional side as we expected worst performance there.

#### Daily living tasks

##### Baking tray task (BTT)

The patient was asked to distribute 16 cubes of 3.5 cm as evenly as possible over a 75 × 100 cm board (as if spreading out buns on a baking tray).^49^ The entire board was scanned using the Microsoft Lens iOS app. Coordinates of all cubes were manually identified using a custom Python script. An average positive x-coordinate indicates a rightward bias.

##### Catherine Bergego scale (CBS)

The CBS is a 10-item observation scale for measuring VSN severity in ADL, and results in a total score of zero (no neglect) to 30 (severe neglect).^50,51^ The CBS was filled out by the patient’s therapist or proxy (partner or caregiver), but we only considered data from forms completed by therapists, as intended. In case <50% of the items of the CBS were observed, the total score was considered not reliable and therefore a missing value.

##### Subjective neglect questionnaire (SNQ)

The SNQ is a 19-item questionnaire for measuring the presence of common problems associated with VSN.^52^ The SNQ is scored on a five-point scale according to the frequency of the occurrence of the difficulty, resulting in a score of 19 (no reported problems) to 95 (many/frequently reported problems).^11^ The SNQ was administered to patients and proxies, but our analysis was based exclusively on forms completed by patients, as intended. In case <50% of the items of the SNQ were filled out, the total score was considered not reliable and therefore a missing value.

### Demographic and injury characteristics

Baseline descriptors were collected, including demographics (age, gender and educational level), stroke characteristics (time post-stroke onset, stroke history, stroke type and lesion side), and global cognitive functioning as measured by the Montreal Cognitive Assessment (MoCA version 8.1).^53^

### Procedure

Eligible patients who met the inclusion criteria were identified by psychologists. After informed consent was given, baseline measurements were performed. Included patients were then randomly assigned to either the experimental or placebo group and received eighteen training sessions spread over 6 weeks (i.e. three sessions per week). The training sessions (including tACS and VST) were offered by the researchers at the patients’ homes. The researchers tested the patients six times on an array of tasks: at baseline (T0), after the first (T1), ninth (T2) and eighteenth (T3) training session, as well as 1 week (T4) and 3 months (T5) after termination of the training. The SCT, CVDT, MLBT-d and SLBT were assessed during all six assessments in the study (T0–T5). The BTT, CBS and SNQ were administered at four assessments (T0, T2, T4 and T5). The SCT, CVDT and MLBT-d were presented on the same touchscreen laptop as was used for the training. PsychoPy was used to control stimulus presentation and recording of behavioral responses.

### Blinding, randomization and treatment allocation

Researchers, therapists and patients were blinded to treatment allocation. We applied minimization as randomization method using MinimPy.^54^ Patients were stratified according to age, gender and having had previous neglect treatment. To double-blind the tACS protocols, the ‘study mode’ of the NeuroConn DC Stimulator (neuroConn GMBH) was implemented using five-digit codes that either initiated the pre-programmed active stimulation protocol or the sham protocol. There was only one unblinded research assistant who assigned a unique code to every enrolled patient and sent the code to the (blinded) researchers who then carried out all other study procedures (including the interventions and assessments). The unblinded research assistant was not further involved in the study. Blinding was removed after data analysis was finalized.

### Statistical analyses

Chi-square (*χ*^2^) and non-parametric Mann–Whitney tests were used to compare demographic and stroke-related characteristics between both groups. Baseline performance on neglect outcome variables was compared with a *t*-test or Mann–Whitney test where appropriate, to detect differences at the start of the trial. To study any associations between the outcome variables, we conducted correlation analyses at T0 and at T5.

To test for change in the primary and secondary outcome measures both within and between groups, linear mixed model regression analysis was used, with a spatial power covariance structure to account for (time-decaying) residual covariance between repeated measures and a random intercept for patients. The predictors of interest were the effects of time and the interaction between time and group (fixed effects). We tested linear, quadratic and cubic effects of time, although conceptually the latter was not expected to be plausible. Gender, age and time since stroke were introduced as potential fixed covariates. A maximum likelihood estimation was used in the process of model selection. We started by focusing on potential removal of higher order interactions between group and time, and higher order effects of time, and finally the covariates. Terms were removed from the model if *P* > 0.05. The coefficients (and their tests) of the final model are reported per outcome measure, based on restricted maximum likelihood estimation. Supplementary *post hoc* contrasts with Bonferroni correction were performed to probe the interaction between time and group by testing differences between groups at specific time-points. As the actual time-points (in days) of measurement vary between patients, we used the mean number of days (across participants) since baseline (T0) to determine the time-points of interest.

The intention-to-treat principle was used by including all patients as randomized in the analyses, regardless of whether they received the complete program. In the context of mixed-model analysis, it is important to note there is no case-wise deletion, but all available data is incorporated. Alpha was set to 0.05. Analyses were performed in IBM SPSS Statistics version 26. Besides the correlation analyses, other analyses were preregistered.^36^

## Results

### Patient characteristics

**Figure 2:**
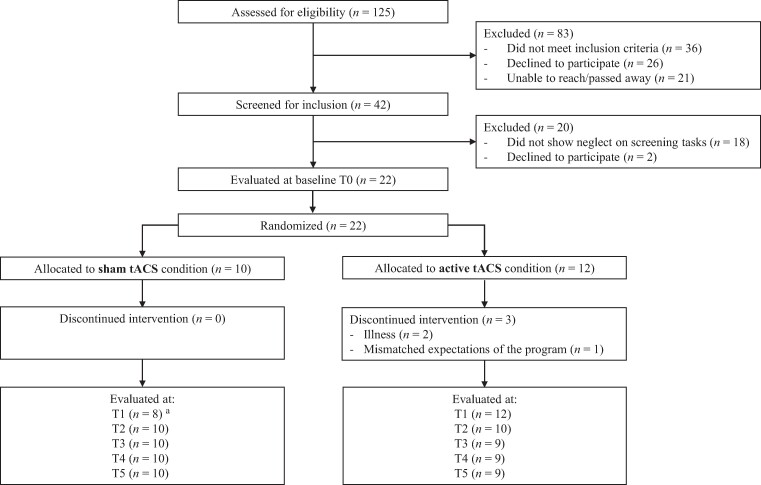
**Patient flow through the study.** Assessments took place before the training (T0, baseline), after the first (T1), ninth (T2) and eighteenth (T3) training session, as well as 1 week (T4) and 3 months (T5) after the end of the training. ^a^Two patients did not perform assessments at T1, due to fatigue. Abbreviation: tACS, transcranial alternating current stimulation.

A total of 125 VSN patients were recruited by the healthcare organizations (Fig. 2). Forty-two patients were screened for inclusion, of whom 22 were included in the study. Supplementary Table 3 depicts the patients’ performances on each screening test on the basis of which they were admitted to the study. The median age of the study sample was 61.0 years and 72.7% (*n* = 16) was male. Of the 22 included patients, 10 were randomly assigned to the sham group and 12 to the active tACS group (Table 1). Three patients in the active tACS group terminated participation prematurely as a result of illness (*n* = 2) or mismatched expectations of the program (*n* = 1), and two patients in the sham group did not perform assessments at T1, both due to fatigue. Figure 2 depicts the remaining number of patients included in each assessment.

**Table 1:** Baseline demographic and stroke- and neglect-related characteristics

	Sham tACS		Active tACS		Comparison
	*n*	Median (IQR)	*n*	Median (IQR)	
Demographics					
Age, years	10	61.00 (12.50)	12	61.00 (20.50)	*U* = 52.00, *z* = −0.528, *P* = 0.597
Gender, % male	10	70	12	75	*P* = 1.000^a^
Educational level, Verhage (0–7)	10	6.00 (1.25)	12	4.50 (2.00)	*U* = 40.50, *z* = −1.389, *P* = 0.165
Stroke characteristics					
Time post-stroke onset, months	10	31.80 (55.82)	12	39.88 (116.86)	*U* = 52.00, *z* = −0.528, *P* = 0.598
Stroke history, % first ever	10	80	12	67	*P* = 0.646^a^
Stroke type, %					*χ* ^2^ = 0.76, *df* = 2, *P* = 0.683
Ischaemic	10	60	12	75	
Intracerebral haemorrhage	10	20	12	17	
Subarachnoid haemorrhage	10	20	12	8	
Stroke side, % right	10	100	12	100	N/A
Neglect side, % left	10	100	12	100	N/A
MoCA (0–30)	10	23.50 (4.75)	12	24.00 (5.50)	*U* = 42.50, *z* = −1.163, *P* = 0.245
Neglect characteristics					
Previous neglect treatment, % yes	10	80	12	83	*P* = 1.000^a^
Neglect variables at baseline					
SCT					
Misses on contralesional side of screen	10	2.50 (19)	12	1.50 (3)	*U* = 49.50, *z* = −0.707, *P* = 0.479
CVDT, weighted hits					
Contralesional condition	10	6.50 (21.48)	11	10.00 (15.01)	*U* = 53.00, *z* = −0.141, *P* = 0.887
Bilateral condition	10	0.50 (11.48)	11	5.00 (10.90)	*U* = 35.00, *z* = 1.437, *P* = 0.151
SNQ (19–95)	10	34.03 (14.50)	11	29.86 (32.99)	*U* = 40.00, *z* = −1.06, *P* = 0.29

		**Mean (SD)**		**Mean (SD)**	

SCT					
QoS for contralesional side of screen	10	0.44 (0.32)	12	0.67 (0.47)	*t*(20) = 1.293, *P* = 0.211
MLBT-d, EWB	10	0.25 (0.18)	12	0.23 (0.18)	*t*(20) = 0.34, *P* = 0.735
SLBT, % deviation of contralesional lines	10	23.93 (20.39)	12	21.30 (14.67)	*t*(20) = 0.35, *P* = 0.729
BTT, mean x-coordinate	10	0.13 (0.11)	11	0.02 (0.08)	*t*(19) = 2.72, *P* = 0.014
CBS (0–30)	7	10.98 (9.13)	6	10.46 (6.94)	*t*(11) = 0.112, *P* = 0.913

^a^Fisher’s exact test (two-tailed) is reported when assumptions of *χ*^2^ have been violated.

Abbreviations: BTT, baking tray task; CBS, Catherine Bergego scale; CVDT, computerized visual detection task; EWB, endpoint weightings bias; IQR, interquartile range; MLBT-d, McIntosh line bisection task-digitized; MoCA, Montreal Cognitive Assessment; N/A, not applicable; QoS, quality of search; SCT, star cancellation task; SLBT, Schenkenberg line bisection task; SNQ, subjective neglect questionnaire.

The two groups were not significantly different with respect to demographic and stroke-related characteristics (all *P* values > 0.165, Table 1). Also, baseline scores on neglect outcome variables were comparable between groups, except for the BTT scores where patients in the active tACS group scored significantly lower (i.e. better performance) at baseline compared to patients in the sham group. Raw mean scores for all assessments (T0–T5) are shown in Supplementary Table 4. Results of the correlation analyses between the outcome variables are shown in Supplementary Table 5.

### Primary outcome: QoS (SCT)

**Figure 3:**
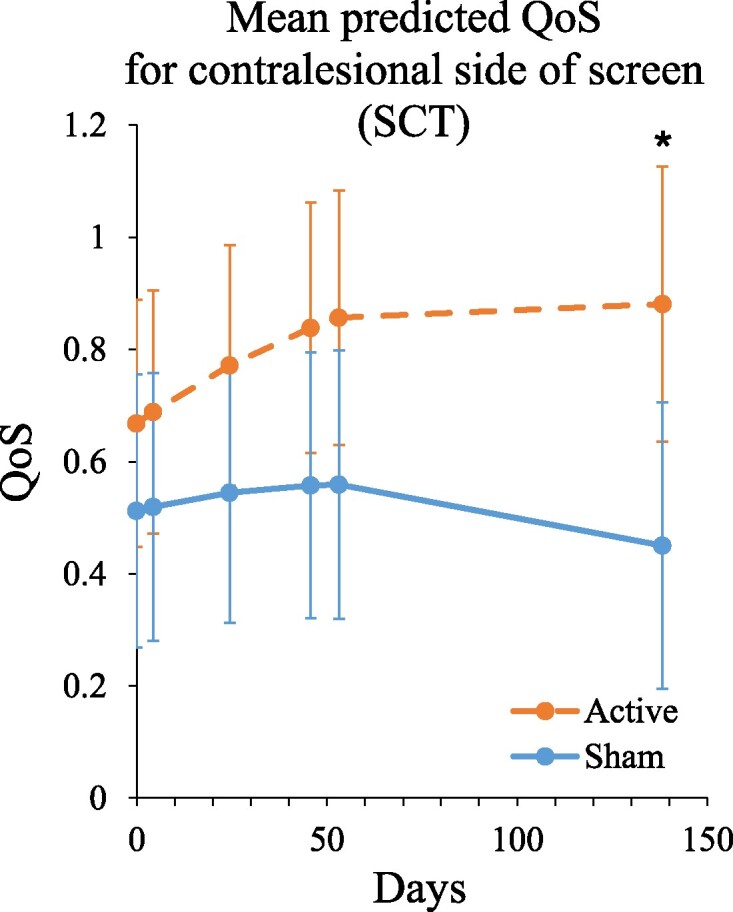
**Mean model-predicted QoS scores for the contralesional side of the screen.** Linear mixed regression analysis including *post hoc* contrasts with Bonferroni correction was performed to probe the interaction between time and group by testing for a difference between active and sham tACS groups at specific time-points. Predicted scores are based on the model that includes linear and quadratic group by time interaction terms. 95% confidence intervals for the mean in the active group (dashed orange line) and the mean in the sham group (solid blue line) are included at the time-points of interest at which the active versus sham contrasts were tested. Higher scores indicate less severe neglect. Asterisks (*) depict significant difference (*P* < 0.05). At Day 138, the active tACS group showed a significantly higher mean QoS score compared to sham (*t*(36) = 2.463, *P* = 0.019). Please note that these test statistics deviate slightly from the test statistics as mentioned in the text, as the predicted scores shown here are based on the regression model that includes linear and quadratic group by time interaction terms, whereas in the text the comparison is based on the final regression model including only a linear group by time interaction term. Abbreviations: QoS, quality of search; SCT, star cancellation task.

We derived the QoS score for the contralesional side of the screen. The final regression model (Table 2) included a linear interaction between group and time (*F*(1, 96) = 5.527, *P* = 0.021), but not a quadratic (*F*(1, 98) = 0.158, *P* = 0.692) nor a cubic (*F*(1, 99) = 2.756, *P* = 0.100) group by time interaction. The significant group by time interaction indicates that the time effect was significantly different between the active group and the sham group (Fig. 3). To conduct supplementary contrast tests of mean treatment differences at specific time-points, the mean number of days (across participants) since baseline was used to determine the time-points (in days) of interest: Day 0 (baseline), Days 4, 24, 46, 53 and 138. These contrasts showed a significant higher mean QoS at Day 138 in the active tACS group compared to sham (*t*(33) = 2.532, *P* = 0.016; *P* values at all other time points were ≥ 0.092). As can be seen in Fig. 3, both groups showed initial improvement in QoS performance (Days 0–53); but in the sham group, this was followed by a decline in scores (Days 53–138). Although further enhancement stagnated, overall, the active group showed significantly more improvement compared to the sham group.

**Table 2:** Final model of fixed-effect predictors and covariates for predicting primary and secondary outcomes

Predictor	*β* ^a^	SE_β_	95% CI lower bound	95% CI higher bound	*P* value
QoS, contralesional side of screen (SCT) across T0 to T5 (*n* = 22)					
Time	0.001	0.001	2.84E-04	0.003	0.016
Group	−0.161	0.155	−0.482	0.160	0.310
Group × time	−0.002	0.001	−0.004	−3.12E-04	0.021
Weighted hits, contralesional condition (CVDT) across T0 to T5 (*n* = 21)					
Time	0.032	0.018	−0.005	0.069	0.088
Group	−1.370	3.322	−8.257	5.517	0.684
Group × time	−0.074	0.026	−0.126	−0.023	0.005
Gender	−9.420	3.482	−16.747	−2.094	0.015
Weighted hits, bilateral condition (CVDT) across T0 to T5 (*n* = 21)					
Time	0.198	0.050	0.098	0.297	<0.001
Time × time	−0.001	3.22E-04	−0.002	−3.06E-04	0.005
Group	−2.130	4.621	−11.750	7.490	0.650
Group × time	−0.071	0.027	−0.124	−0.017	0.010
Gender	−14.148	4.977	−24.581	−3.715	0.011
EWB (MLBT-d) across T0 to T5 (*n* = 22)					
Time	−0.002	0.001	−0.003	−2.92E-04	0.018
Time × time	1.08E-05	4.51E-06	1.77E-06	1.99E-05	0.020
Relative deviation on contralesional lines (SLBT) across T0 to T5 (*n* = 22)					
Time	−0.047	0.020	−0.088	−0.007	0.023
Mean x-coordinate (BTT) at T0, T2, T4 and T5 (*n* = 21)					
Group	0.078	0.030	0.014	0.142	0.019
CBS at T0, T2, T4 and T5 (*n* = 13)					
Time	−0.134	0.051	−0.239	−0.030	0.014
Time × time	0.001	3.47E-04	3.89E-05	0.001	0.040
SNQ at T0, T2, T4 and T5 (*n* = 22)					
Time	−0.233	0.066	−0.366	−0.101	0.001
Time × time	0.001	4.39E-04	4.93E-04	0.002	0.003

^a^ *β* coefficients are shown in reference to the active group.

Abbreviations: BTT, baking tray task; CBS, Catherine Bergego scale; CI, confidence interval; CVDT, computerized visual detection task; EWB, endpoint weightings bias; MLBT-d, McIntosh line bisection task-digitized; QoS, quality of search; SCT, star cancellation task; SLBT, Schenkenberg line bisection task; SNQ, subjective neglect questionnaire.

For the sake of completeness, we conducted a second analysis for the ipsilesional side of the screen, revealing no group by time interaction (*P* values for linear, quadratic and cubic functions ≥ 0.655). This outcome aligns with the underlying theory of our interventional approach, which predicts that contralesional stimulation does not affect (or negatively affects) performance in the ipsilesional side compared to sham stimulation. The final model is depicted in Supplementary Table 6.

**Figure 4:**
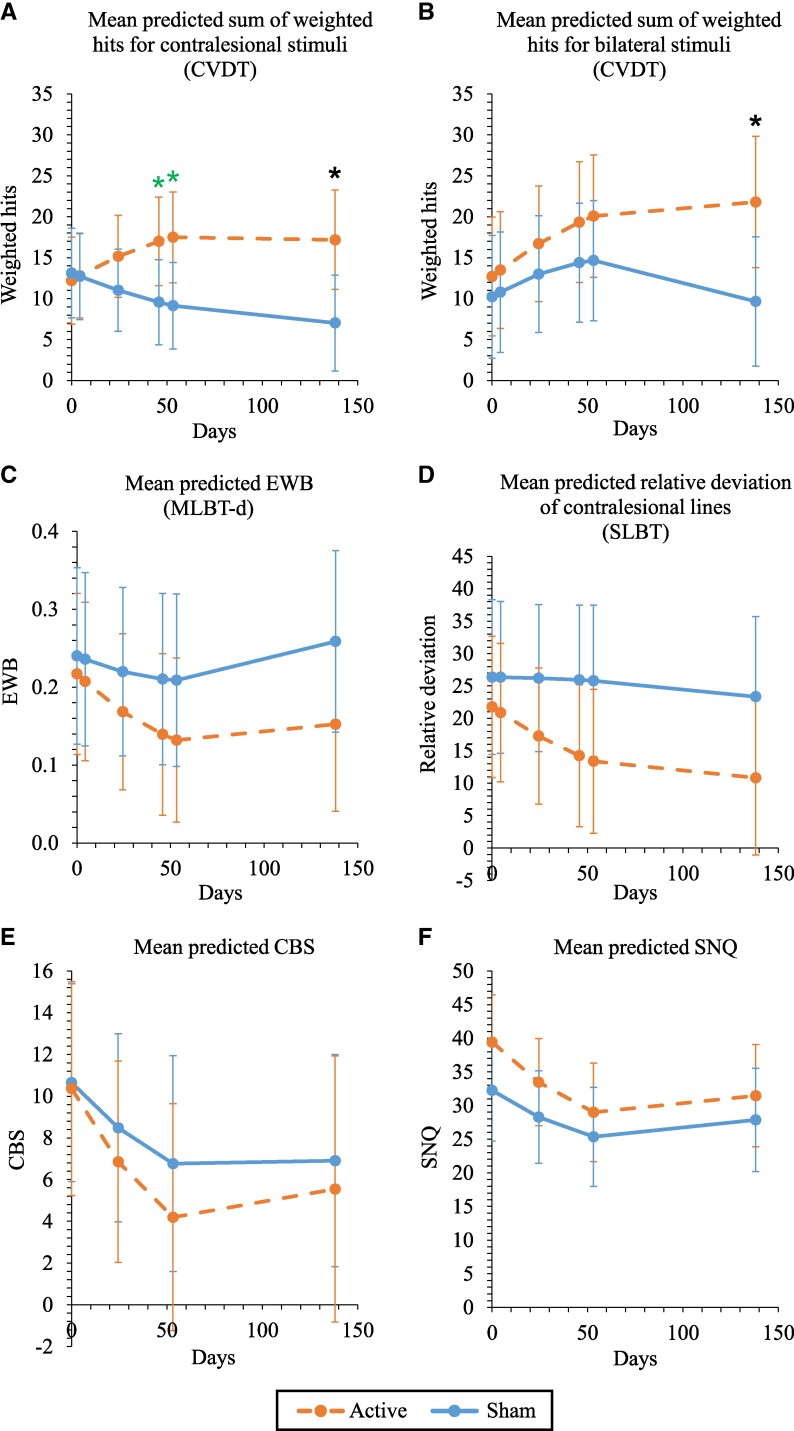
**Mean model-predicted scores of secondary outcomes.** Linear mixed regression analysis including *post hoc* contrasts with Bonferroni correction was performed to probe the interaction between time and group by testing for a difference between active and sham tACS groups at specific time-points. Predicted scores of **(A)** sum of weighted hits for contralesional stimuli, **(B)** sum of weighted hits for bilateral stimuli, **(C)** EWB, **(D)** relative deviation of contralesional lines, **(E)** CBS and **(F)** SNQ, are based on the models that include linear and quadratic group by time interaction terms. 95% confidence intervals for the mean in the active group (dashed orange line) and the mean in the sham group (solid blue line) are included at the time-points of interest at which the active versus sham contrasts were tested. Neglect is less severe when scores are higher (CVDT), or closer to zero (EWB, SLBT), or lower (CBS, SNQ). Asterisks (*) depict significant difference (*P* < 0.05). The active tACS group showed a significantly higher mean visual detection performance in the contralesional condition of the CVDT **(A)** at Day 46 (*t*(25) = 2.146, *P* = 0.042), at Day 53 (*t*(27) = 2.343, *P* = 0.027) and at Day 138 (*t*(43) = 2.514, *P* = 0.016), as well as in the bilateral condition of the CVDT **(B)** at Day 138 (*t*(34) = 2.288, *P* = 0.028). Please note that test statistics presented here deviate slightly from the test statistics as mentioned in the text, as the predicted scores shown here are based on the regression model that includes linear and quadratic group by time interaction terms, whereas in the text the comparisons are based on the final regression models including only a linear group by time interaction term. Asterisks in green only apply to the regression model that includes both linear and quadratic group by time interaction terms, but do not apply to the regression model that includes only a linear group by time interaction term. Abbreviations: CBS, Catherine Bergego scale; CVDT, computerized visual detection task; EWB, endpoint weightings bias; MLBT-d, McIntosh line bisection task-digitized; SLBT, Schenkenberg line bisection task; SNQ, subjective neglect questionnaire.

Note that Figs 3 and 4 plot model-predicted means, and not observed means, since the actual time-points (days) of measurement vary between patients (i.e. unbalanced longitudinal data).

### Secondary outcomes

#### Sum of weighted hits (CVDT)

One patient of the active tACS group displayed a very high variability of weighted hits scores and was identified as statistical outlier (> 3.0*IQR from Q1 and Q3), thus the data presented here includes a total sample of 21 patients. As expected, the baseline scores were significantly lower (i.e. worse performance) of the contralesional and bilateral stimulus conditions compared to baseline scores of the ipsilesional condition (contralesional versus ipsilesional: *Z* = 4.02, *P* < 0.001; bilateral versus ipsilesional: *Z* = 3.980, *P* < 0.001).

The final model of the contralesional condition (Table 2) included a linear group by time interaction (*F*(1, 52) = 8.493, *P* = 0.005), but not a quadratic (*F*(1, 44) = 3.760, *P* = 0.059) nor a cubic (*F*(1, 78) = 0.913, *P* = 0.342) group by time interaction. Furthermore, only gender was included as a covariate (*F*(1, 18) = 7.319, *P* = 0.015). The significant group by time interaction indicates that the time effect was different between the active and sham group (Fig. 4A); there was a positive linear effect of time in the active group (*P* = 0.088), and a negative linear effect of time in the sham group (*P* = 0.020). Follow-up contrasts showed a significant higher mean visual detection performance at Day 138 in the active compared to the sham group (*t*(40) = 2.933, *P* = 0.006; *P* values at all other time-points were ≥ 0.108).

The final model of the bilateral condition (Table 2) included a linear group by time interaction (*F*(1, 68) = 6.940, *P* = 0.010), but not a quadratic (*F*(1, 60) = 0.086, *P* = 0.770) nor a cubic (*F*(1, 82) = 1.194, *P* = 0.278) group by time interaction. Also, there was a negative quadratic effect of time (*F*(1, 55) = 8.739, *P* = 0.005), and gender was included as a covariate (*F*(1, 19) = 8.082, *P* = 0.011). The significant group by time interaction indicates that the time effect was different between the active and sham group. There was an initial improvement in both groups (Days 0–53; Fig. 4B), but in the sham group this was followed by a decline in scores (Days 53–138). Additional contrasts showed significant better mean performance at Day 138 in the active compared to the sham group (*t*(32) = 2.283, *P* = 0.029; *P* values at all other time-points were ≥ 0.208).

For the sake of completeness, we conducted a final analysis for the ipsilesional condition, revealing no group by time interaction (*P* values for linear, quadratic and cubic functions ≥ 0.459). Again, this outcome aligns with the underlying theory of our interventional approach, which predicts that contralesional stimulation does not affect (or negatively affects) visual detection performance in the ipsilesional hemifield compared to sham stimulation. The final model is depicted in Supplementary Table 6.

#### EWB (MLBT-d)

The final model of the EWB (Table 2) did not include any group by time interaction (linear: *F*(1, 78) = 2.610, *P* = 0.110; quadratic: *F*(1, 58) = 0.314, *P* = 0.577; cubic: *F*(1, 88) = 3.354, *P* = 0.070). The final model included not only a linear main effect of time (*F*(1, 50) = 5.954, *P* = 0.018), but also a quadratic main effect of time (*F*(1, 54) = 5.744, *P* = 0.020). Figure 4C shows that, over the course of 6 weeks training and one-week follow-up (Days 0–53), regardless of whether tACS was involved or not, patients showed less bias towards the right endpoints of the lines; however, subsequent to that period (Days 53–138), any further improvement stagnated (active group) or even reversed (sham group).

#### Relative deviation (SLBT)

As expected, the baseline relative deviation was significantly higher (i.e. worse performance) of the lines positioned contralesional compared to lines either positioned in the middle or ipsilesional (contralesional versus middle: *Z* = 3.652, *P* < 0.001; contralesional versus ipsilesional: *t*(21) = 4.205, *P* < 0.001). The final model of the contralesional lines (Table 2) included no group by time interaction (linear: *F*(1, 82) = 1.730, *P* = 0.192; quadratic: *F*(1, 68) = 1.751, *P* = 0.190; cubic: *F*(1, 92) = 2.529, *P* = 0.115), and included only a linear main effect of time (*F*(1, 81) = 5.348, *P* = 0.023). This means that, over the course of time (Days 0–138), regardless of whether patients received tACS, patients showed less bias towards the ipsilesional side of the lines (Fig. 4D).

#### Mean x-coordinate (BTT)

One patient of the active tACS group did not understand the instructions of the BTT, and was excluded from the BTT analyses. The final model (Table 2) did not include a group by time interaction (linear: *F*(1, 58) = 0.487, *P* = 0.488; quadratic: *F*(1, 56) = 2.431, *P* = 0.125; cubic: *F*(1, 57) = 0.479, *P* = 0.492), indicating that patterns of effects over time were similar for both groups. Furthermore, no time effect was found (linear, quadratic and cubic; all *P* values > 0.602).

#### CBS

In eight out of 22 patients (36%), there was no therapist involved in the patient’s care to fill out the CBS. Of the remaining fourteen patients (64%), there were seven forms that were not reliable (i.e. < five valid items), four missing forms due to practical concerns (such as therapist on leave or employed elsewhere), and five missing forms due to dropout. This eventually led to a sample size of thirteen patients (59%; seven sham group, six active group), with a mean number of filled out forms per patient of 3.08 (SD = 1.12).

The final model for the CBS (Table 2) did not include a group by time interaction (linear: *F*(1, 27) = 0.063, *P* = 0.804; quadratic: *F*(1, 28) = 0.417, *P* = 0.524; cubic: *F*(1, 28) = 0.183, *P* = 0.672), indicating that the time effect was similar for both groups (Fig. 4E). There was, however, evidence not only for a linear main effect of time (*F*(1, 26) = 6.948, *P* = 0.014), but also a quadratic main effect of time (*F*(1, 26) = 4.706, *P* = 0.040). Over the course of 6 weeks training, regardless of whether tACS was involved or not, patients showed less VSN behavior in daily life activities (Days 0–53); however, subsequent to that period, any further enhancement in effects seemed to stagnate (Days 53–138; Fig. 4E).

#### SNQ

Besides the eight forms that were missing due to the three patients that terminated participation prematurely (dropouts), there was only one form that was not reliable (i.e. < 10 valid items). Thus, sample size remained at 22, with a mean number of filled out forms per patient of 3.59 (SD = 0.96).

The final model for the SNQ (Table 2) did not include a group by time interaction (linear: *F*(1, 55) = 0.494, *P* = 0.485; quadratic: *F*(1, 54) = 0.341, *P* = 0.562; cubic: *F*(1, 54) = 0.002, *P* = 0.965), indicating that the time effect was similar for both groups (Fig. 4F). As was seen for the CBS, there was evidence not only for a linear main effect of time (*F*(1, 53) = 12.741, *P* = 0.001), but also a quadratic main effect of time (*F*(1, 52) = 9.989, *P* = 0.003). Figure 4F shows that, during the training trajectory of 6 weeks, regardless of stimulation group, patients experienced less problems due to VSN in their daily lives (Days 0–53); however, this initial phase of progress was followed by a plateau in effects (Days 53–138).

## Discussion

This study evaluated the additive effects of multi-session tACS at alpha frequency, combined with VST, on alleviating attention deficits in chronic stroke patients suffering from VSN. We found that patients receiving active tACS with VST showed a significantly stronger improvement in their visual search performance on the contralesional side measured with a computerized cancellation task (SCT, primary outcome measure), as compared to patients receiving sham (placebo) stimulation with VST. Additionally, our novel tACS approach resulted in significantly stronger improvements in the allocation of attention towards the contralesional side measured with a computerized visual detection paradigm (CVDT), also compared to sham stimulation. Furthermore, although no differences in performance were found between active and sham tACS on the line bisection tasks (MLBT-d and SLBT) and the measures of neglect behavior in basic ADL (BTT, CBS and SNQ), significant time-dependent improvements were observed, emphasizing the potential for recovery through rehabilitation in the later phases following a stroke.

Our findings closely parallel those of our prior single-session tACS study in subacute stroke patients.^31^ There too, improvements were found specific to active tACS on a cancellation task (bells task) and the same visual detection task, but not on a line bisection task. The repeatedly observed divergent effects are likely due to the varying cognitive demands of distinct tasks. For example, cancellation tasks require visual search and tap into a different type of cognitive process than line bisection tasks.^55,56^ Cancellation tasks may therefore better correspond with the skills and cognitive processes trained by VST, and, consequently, are more likely to capture the accurate underlying cognitive process.

Our current results are the first to show that multi-session tACS complemented with VST leads to long-term benefits of up to three months post treatment. Stimulation effects were seen in QoS on the contralesional side and in visual detection in the contralesional and bilateral conditions. The bilateral condition directly relates to the visual extinction phenomenon, a neurological syndrome closely associated with VSN. Extinction is characterized by the failure to process or attend to a contralesional event when a second competing stimulus is simultaneously presented in the ipsilesional hemifield.^57,58^ These results suggest that our tACS therapy enhances perception in the neglected side, also/even in the presence of distractors in the non-neglected side. Additionally, this enhancement of attention in the neglected side was not accompanied by an impairment of attention in the non-neglected side, because stimulation did not affect performance in the ipsilesional side/condition of the SCT and CVDT, as may be the case when simply reducing cortical excitability of the contralesional hemisphere using conventional NIBS approaches.^59,60^

No tACS effects were found on questionnaires/tasks requiring more dynamic interactions (i.e. ADL-related measures: BTT, CBS and SNQ). This may be because ADL measures do not, typically, assess the ‘efficiency’ with which daily-life activities are carried out, but merely measure the severity or frequency of occurrence of neglect-related behavior. In this sense, these measures are different from the SCT and CVDT where a time component or time restriction is included, and are less capable of detecting changes in performance efficiency, such as a better quality or effectiveness of the process to perform a daily life activity (e.g. less steps needed) or less time needed to complete an activity. Another explanation for the lack of a generalization effect could be the digitalized format of the VSN training. Possibly, training on a computer screen does not affect daily life tasks. Nonetheless, significant time-dependent improvements were observed on the CBS and SNQ, irrespective of stimulation group, suggesting that patients implemented the acquired visual scanning strategies of the VST in daily life. The digitized VST brings advantages as it is easily usable on a touchscreen and adapts to the patient’s performance ability. Also, the training program encompasses a variety of engaging tasks and provides data-driven feedback, ensuring a varied and stimulating experience to foster commitment and adherence among patients. Results of the CBS should, however, be interpreted with caution as sample size was reduced to a mere thirteen patients (59% of 22), reducing statistical power and increasing the likelihood of Type II errors.

The lack of stimulation effects on measures of ADL could have (also) been caused by the large performance variability seen in our patient sample. Patients were included based on the presence of VSN symptoms on several conventional, paper-and-pencil neglect tests (not including any measure of ADL), which, evidently, does not necessarily imply that they (also) suffered from neglect in dynamic daily living situations. Indeed, dissociations have been found between patients who displayed symptoms of VSN on conventional, static measures but not on measures of daily functioning, and vice versa.^9,61,62^

Overall, assessing the transfer of treatment effects to daily life remains a considerable challenge. For instance, tools used to evaluate ADL can be significantly influenced by other VSN-related issues; motor deficits, for instance, have demonstrated notable effects on measures like the Barthel index or functional independence measure.^9^ Additionally, both the quantity and duration of treatment sessions can influence effectiveness. There remains a gap in the literature regarding systematic exploration of the optimal combination of treatment intensity and duration necessary for effective transfer of treatment effects to daily life.^9,13^

### Shortcomings and strengths

A limitation is the study’s response rate of 17.6% (22 included patients out of 125 eligible patients), which may raise concerns about the generalizability of findings to the broader neglect population. The low response rate may have been the result of the rather strict criteria that we use for research purposes while the clinical stroke population is much more diverse. Also, some patients were deceased or were unreachable since their discharge from rehabilitation.

A second limitation regards the novel approach for administering and analyzing the line bisection task (i.e. ‘endpoint weightings analysis’) that has recently been proposed by McIntosh *et al.*^46,47^ The EWB, representing the lateral attentional bias, has proven to be more sensitive to right-sided brain damage than the ‘classical’ bisection error, and relates more strongly to cancellation and copying measures.^46^ However, also on this new, more sensitive measure we did not observe tACS effects in the current study. We speculate that the means of assessment employed in this study may not have been the optimal choice; the ‘touch’ of the finger on a touchscreen laptop may have resulted in a less precise bisection mark compared to when the mark would be placed with pencil on paper (as was done in McIntosh *et al.*^46^) or even with pencil on touchscreen laptop or tablet.

While we did not explicitly assess patients’ ability to distinguish between active and sham tACS, it is important to note that tACS does not generate audible signals and somatosensory sensations during active stimulation.^63^ Furthermore, we included a ramp-up period in both conditions so patients could (potentially) perceive the onset of stimulation; however, in the sham condition, this was followed by a ramp-down phase after a brief interval. Blinding effectiveness has previously been demonstrated in comparable studies involving healthy volunteers, utilizing identical tACS devices and stimulation parameters.^24,25,64^

Several important strengths of this study are in reference to the double-blind, randomized controlled study design. All patients, researchers and therapists were blinded to treatment allocation, and the outcomes of the assessments did not affect therapists in any way. Furthermore, we used minimization as randomization method, to ensure balance across important patient characteristics that could have affected the study outcomes. Lastly, we adopted an interdisciplinary approach where brain-based NIBS is combined with behavior-based rehabilitation techniques combined with function-based and clinically relevant outcome measures, both in the short term and the long term.

### Future research and clinical applications

The combined tACS-VST approach should be further tested in a rehabilitation setting, with subacute patients, and explore patterns of recovery within specific patient profiles. For instance, VSN involves different clinical subtypes that vary in frame of reference (egocentric and allocentric), sensory modality (visual, auditory, haptic and tactile) and region of space (personal, peripersonal and extrapersonal),^65-67^ and different clinical subtypes have been associated with different lesion sites.^68,69^ Evaluating at subgroup level, or even at individual level, with due consideration for distinct clinical subtypes and lesion location, will bring to light which patients are likely to benefit (most) from the treatment. Also, it is necessary to explore the most cost-effective setting for implementing the intervention. For example, although VST is traditionally offered in the clinical setting, our digitized VST program, in combination with a portable, low-cost tACS, would lend well to be used in a home-setting.^70^

As our VST composed of both bottom–up and top–down strategies, it remains unclear what component of the training induces the strongest neuronal plasticity when combined with tACS. Determining the most influential element, whether it is the strengthening of exogenous orienting towards external cues (associated with the ventral attention network^71,72^) or the enhancement of systematic learning by top–down mechanisms (linked to endogenous attention regulated by the dorsal attention network^71,72^), warrants further exploration.

Regarding optimization of the tACS protocol itself, significant efforts have been made towards individualized stimulation parameters (e.g. personalized stimulation montage/location, dose and waveform) using individual brain morphology (with computational head modeling) and neuroimaging (with EEG and fMRI).^17^ For example, we demonstrated in healthy individuals that stimulation at intrinsic individual frequencies, compared to stimulation at flanker frequencies leads to larger alpha power lateralization after stimulation.^25^ Personalizing tACS frequencies to individual brain rhythms could indeed improve tACS efficacy in a healthy population group,^73^ yet how such approach would be most effectively implemented to work in clinical populations where brain rhythms are disrupted after brain damage,^74,75^ is clearly less straightforward and should be addressed through forthcoming research.

## Conclusion

In conclusion, multi-session tACS at alpha frequency complemented with VST, led to significantly stronger improvement in visual search performance and more enhanced perception in the neglected side in chronic stroke patients with VSN up to three months post treatment, compared to sham tACS with VST. While we did not find additive effects of stimulation on other measures (line bisection and ADL), it is noteworthy that time-dependent improvements on all but one of these measures were observed, regardless of stimulation group. Future research should focus on specific clinical neglect profiles to account for the heterogeneous nature of the neglect syndrome, and create stimulation protocols customized for VSN patient groups to allow enhanced tACS efficacy, that ultimately transfers to beneficial effects in patients’ daily living.

## Supplementary Material

fcae287_Supplementary_Data

## Data Availability

The data that support the findings of this study are available from the corresponding author, upon reasonable request. The codes that were used for statistical analyses are provided within the supplementary material (Supplementary Table 7).
