# Supplementary material for: Alpha transcranial alternating current stimulation as add-on to neglect training: a randomized trial
Source: Brain Commun. 2024 Aug 30;6(5):fcae287. doi: 10.1093/braincomms/fcae287 (PMC11411215; doi:10.1093/braincomms/fcae287)
Supplement: fcae287_Supplementary_Data [file fcae287_supplementary_data.pdf]

Supplementary Table 1 Cut-off criterion per screening test

Screening test	Cut-off criterion
BT[1]	Four or more total omissions and an asymmetry of three or more between left and right hemifields.
BB[2]	Total score of less than seventeen and a laterality of less than 45 percent.
SLBT[3]	Bisection mark deviations of ten percent or more.
MLBT[4–6]	EWB of more than 0.09 and less than −0.13 for left and right VSN, respectively.

Patients were included if performance deviated from normal range on minimally one of the four following screening tests (all administered on paper): BT, BB, SLBT, MLBT. More detail about the screening tests can be found in the protocol publication.[6]
Abbreviations: BB, balloons-subtest B; BT, bells task; EWB, endpoint weightings bias; MLBT, McIntosh line bisection task; SLBT, Schenkenberg line bisection task; VSN, visual spatial neglect.

Supplementary Table 2 Formula(s) per outcome measure

Outcome measure	Formula
SCT[7]	$QoS = \frac{{N_{cor}}^2}{N_{tar} \cdot t_{tot}}$<br><br>Where:<br>$QoS$ is the quality of search score;<br>N_{cor} is the number of cancelled targets (correct responses);<br>N_{tar} is the total number of targets;<br>t_{tot} is the total time spent.
CVDT[8]	$weighted\ hits = \frac{\log_{10} contrast_{max}}{\log_{10} contrast_{trial}}$<br><br>Where:<br>$contrast_{max}$ is 100%.
MLBT-d[4–6]	The lines have the following lengths and spatial positions in relation to the midpoint of the screen: Line A (-40 mm to 40 mm), Line B (-80 mm to +40 mm), Line C (-40 mm to +80 mm) and Line D (-80 mm to +80 mm). The center of the laptop screen is aligned with the patient's vertical body midline. The screen presents a single line at a time to the patient, who is instructed to bisect the line by marking a position (P). In the endpoint weightings analysis, the position of the patient's response and of the left and right endpoints are coded as horizontal coordinates relative to the midline of the screen. The analysis then focuses on how this response position varies from trial-to-trial as a consequence of changes in the left endpoint (lines A & C vs B & D) and changes in the right endpoint (lines A & B vs C & D). Perfect performance would yield symmetrical right and left endpoint weightings of 0.5, and an EWB value of zero.<br><br>$dPL = \frac{(P_{mean} \text{ in line A and C}) - (P_{mean} \text{ in line B and D})}{40}$<br><br>$dPR = \frac{(P_{mean} \text{ in line C and D}) - (P_{mean} \text{ in line A and B})}{40}$<br><br>$EWB = dPR - dPL$<br><br>Where:<br>$P$ is the position of the patient's response;<br>dPL is the left endpoint weighting;<br>dPR is the right endpoint weighting;<br>dPL and dPR are expressed as a proportion of the endpoint change (40 mm), and range from 0 to 1;<br>EWB is the endpoint weightings bias (i.e. bias towards one of the two endpoints).
SLBT[3]	$deviation = \frac{(bisection\ mark - true\ center)}{true\ center} * 100\%$<br><br>Where:<br>values are always measured from the left end of the line.

Abbreviations: CVDT, computerized visual detection task; MLBT-d, McIntosh line bisection task-digitized; SCT, star cancellation task; SLBT, Schenkenberg line bisection task.

Supplementary Table 3 Overview of performance per screening test of included patients (*n* = 22)

Patient	BT	BB	SLBT	MLBT
9	0	0	1	1
11	0	1	1^{X}	1^{R}
13	1^{BL}	1^{R}	1	1
2	1	1	1	1
16	1	1^{BL}	1	0
14	1	1	missing	1
6	1	1	1^{BL}	missing
19	1	1	1^{BL}	1
17	0	0	1^{X}	0
15	1	0	1	1
18	1	1	1	0
22	0	1	1^{BL}	1
21	0	0	1	0
10	1	0	1	0
4	0	0	1	1
5	0	0	0	1
7	0	1^{BL}	1	1
8	missing	0	1	1
12	1^{BL}	1	1	1
3	0	0	1^{X}	1
20	1	0	1	1
1	1	1	1	1

1 = scoring above cut-off, implying an indication of VSN; 0 = scoring below cut-off, implying no indication of VSN. Grey cells imply a right-sided bias to an extent that is indicative of left-sided VSN, on the basis of which patients were included in the study. Please note that only included patients (with right-hemispheric lesions) are shown in this table. Although we also screened patients with left-hemispheric lesions, none scored above cut-off, thus only patients with right-hemispheric lesions were included in the study. On average, mild VSN was detected on conventional neuropsychological tests, known for their notorious insensitivity in measuring VSN during the chronic phase.

BT and BB: Lateralized omissions were calculated for the BT and BB. Distribution of omissions is indicated with superscript R in case of a right-sided distribution to an extent that is indicative of right-sided VSN, and with superscript BL in case of a bilateral distribution.

SLBT and MLBT: The relative deviation score and EWB were calculated for the SLBT and MLBT, respectively. Bias is indicated with a superscript X in case the bisection mark was placed ipsilesional (indeed implying right-sided bias) but – unusually – *not on the left lines*. (One would generally expect most bias on left-sided lines in left-sided VSN. Relative deviation on left lines is used as a secondary outcome measure of the SLBT in this study.) Furthermore, superscript R indicates that the bisection mark was placed contralesional implying right-sided VSN, and superscript BL indicates that both ipsilesional and contralesional biases were shown.

Abbreviations: BB, balloons-subtest B; BT, bells task; MLBT, McIntosh line bisection task; SLBT, Schenkenberg line bisection task; VSN, visual spatial neglect.

Supplementary Table 4 Mean (SD) neglect scores per assessment

	T0 Baseline		T1 After 1st session		T2 After 9th session		T3 After 18th session		T4 1-week follow-up		T5 3-month follow-up	
	n	Mean (SD)	*n*	Mean (SD)	*n*	Mean (SD)	*n*	Mean (SD)	*n*	Mean (SD)	*n*	Mean (SD)
**SCT, QoS contralesional**												
Active	12	0.67 (0.47)	12	0.71 (0.46)	10	0.73 (0.39)	9	0.82 (0.50)	9	0.96 (0.67)	9	0.90 (0.52)
Sham	10	0.44 (0.32)	8	0.63 (0.25)	10	0.60 (0.28)	10	0.52 (0.23)	10	0.56 (0.37)	10	0.45 (0.21)
**CVDT, weighted hits contralesional**												
Active	11	11.38 (11.05)	11	9.91 (10.20)	9	11.53 (10.77)	8	14.56 (11.51)	8	16.51 (11.92)	8	15.21 (8.00)
Sham	10	11.14 (11.19)	8	10.74 (10.49)	10	9.69 (8.63)	9	8.14 (6.81)	10	7.12 (7.77)	9	5.22 (4.69)
**CVDT, weighted hits bilateral**												
Active	11	9.41 (10.96)	11	11.41 (12.24)	9	11.16 (14.37)	8	16.99 (17.57)	8	18.49 (16.49)	8	18.87 (16.76)
Sham	10	6.34 (11.03)	8	9.96 (11.32)	10	11.69 (12.75)	9	12.67 (12.90)	10	11.08 (14.05)	9	7.84 (8.52)
**MLBT-d, EWB**												
Active	12	0.23 (0.18)	12	0.22 (0.18)	10	0.14 (0.10)	9	0.10 (0.14)	9	0.12 (0.12)	9	0.12 (0.14)
Sham	10	0.25 (0.18)	9	0.16 (0.13)	10	0.23 (0.19)	10	0.22 (0.17)	10	0.21 (0.22)	10	0.26 (0.24)
**SLBT, % deviation on contralesional lines**												
Active	12	21.30 (14.67)	12	22.21 (18.38)	10	16.26 (9.85)	9	21.51 (16.73)	9	8.94 (6.18)	9	11.61 (5.31)
Sham	10	23.93 (20.39)	8	28.32 (26.62)	10	32.72 (30.60)	10	24.97 (21.62)	10	22.04 (19.67)	10	23.80 (24.63)
**BTT, average x-coordinate**												
Active	11	0.02 (0.08)	-	-	9	0.02 (0.06)	-	-	8	0.04 (0.09)	8	0.02 (0.12)
Sham	10	0.13 (0.11)	-	-	10	0.13 (0.19)	-	-	10	0.06 (0.07)	10	0.10 (0.07)
**CBS**												
Active	6	10.46 (6.94)	-	-	5	5.98 (4.06)	-	-	4	3.61 (3.06)	3	4.07 (5.25)
Sham	7	10.98 (9.13)	-	-	5	7.57 (3.76)	-	-	4	5.44 (6.35)	6	5.83 (4.79)
**SNQ**												
Active	11	40.30 (17.18)	-	-	9	29.28 (9.46)	-	-	9	28.83 (12.20)	9	28.79 (8.75)
Sham	10	33.52 (13.70)	-	-	10	25.57 (4.43)	-	-	10	26.98 (8.78)	10	27.67 (10.40)

Abbreviations: BTT, baking tray task; CBS, Catherine Bergego scale; CVDT, computerized visual detection task; EWB, endpoint weightings bias; MLBT-d, McIntosh line bisection task-digitized; QoS, quality of search; SCT, star cancellation task; SD, standard deviation; SLBT, Schenkenberg line bisection task; SNQ, subjective neglect questionnaire.

Supplementary Table 5 Correlation analysis between the values of the visuospatial neglect tests across patients at T0 (A) and at T5 (B)

A. Pearson correlations across patients at T0

		QoS (contralesional; SCT)	Weighted hits (contralesional; CVDT)	Weighted hits (bilateral; CVDT)	EWB (MLBT-d)	Relative deviation (SLBT)	Mean x-coordinate (BTT)	CBS	SNQ
**QoS (contralesional; SCT)**	Pearson Correlation	1							
	Sig. (2-tailed)								
	N	22							
**Weighted hits (contralesional; CVDT)**	Pearson Correlation	0.379	1						
	Sig. (2-tailed)	0.091							
	N	21	21						
**Weighted hits (bilateral; CVDT)**	Pearson Correlation	0.414	0.768	1					
	Sig. (2-tailed)	0.062	**<0.001**						
	N	21	21	21					
**EWB (MLBT-d)**	Pearson Correlation	-0.300	-0.517	-0.508	1				
	Sig. (2-tailed)	0.176	**0.016**	**0.019**					
	N	22	21	21	22				
**Relative deviation (SLBT)**	Pearson Correlation	-0.215	-0.325	-0.333	0.430	1			
	Sig. (2-tailed)	0.336	0.151	0.140	**0.046**				
	N	22	21	21	22	22			
**Mean x-coordinate (BTT)**	Pearson Correlation	-0.382	-0.311	-0.323	0.329	0.478	1		
	Sig. (2-tailed)	0.088	0.182	0.164	0.145	**0.028**			
	N	21	20	20	21	21	21		
**CBS**	Pearson Correlation	-0.669	-0.465	-0.469	0.522	0.169	0.184	1	
	Sig. (2-tailed)	**0.012**	0.109	0.106	0.067	0.581	0.547		
	N	13	13	13	13	13	13	13	
**SNQ**	Pearson Correlation	-0.261	-0.346	-0.235	0.384	0.142	0.324	0.222	1
	Sig. (2-tailed)	0.254	0.136	0.318	0.085	0.539	0.163	0.487	
	N	21	20	20	21	21	20	12	21

B. Pearson correlations across patients at T5

		QoS (contralesional; SCT)	Weighted hits (contralesional; CVDT)	Weighted hits (bilateral; CVDT)	EWB (MLBT-d)	Relative deviation (SLBT)	Mean x-coordinate (BTT)	CBS	SNQ
**QoS (contralesional; SCT)**	Pearson Correlation	1							
	Sig. (2-tailed)								
	N	19							
**Weighted hits (contralesional; CVDT)**	Pearson Correlation	0.527	1						
	Sig. (2-tailed)	**0.030**							
	N	17	17						
**Weighted hits (bilateral; CVDT)**	Pearson Correlation	0.263	0.696	1					
	Sig. (2-tailed)	0.307	**0.002**						
	N	17	17	17					
**EWB (MLBT-d)**	Pearson Correlation	-0.373	-0.261	-0.365	1				
	Sig. (2-tailed)	0.116	0.312	0.150					
	N	19	17	17	19				
**Relative deviation (SLBT)**	Pearson Correlation	-0.283	-0.509	-0.459	0.458	1			
	Sig. (2-tailed)	0.241	**0.037**	0.064	**0.048**				
	N	19	17	17	19	19			
**Mean x-coordinate (BTT)**	Pearson Correlation	-0.605	-0.494	-0.248	0.271	0.272	1		
	Sig. (2-tailed)	**0.008**	0.052	0.355	0.276	0.275			
	N	18	16	16	18	18	18		
**CBS**	Pearson Correlation	-0.520	-0.085	0.111	-0.006	0.261	-0.006	1	
	Sig. (2-tailed)	0.152	0.828	0.775	0.987	0.498	0.987		
	N	9	9	9	9	9	9	9	
**SNQ**	Pearson Correlation	-0.109	0.030	0.479	0.264	-0.061	-0.112	0.619	1
	Sig. (2-tailed)	0.657	0.908	0.052	0.275	0.806	0.657	0.075	
	N	19	17	17	19	19	18	9	19

A. At T0, the primary outcome variable (QoS; contralesional side; SCT) correlated significantly with the CBS score (-0.669, p = 0.012). A negative correlation between the SCT and CBS is to be expected as a higher QoS and a lower CBS indicate better performance. **B.** At T5, QoS correlated significantly with the weighted hits (contralesional condition; CVDT) (0.527, p = 0.030) and the mean x-coordinate (BTT) (-0.605, p = 0.008). A negative correlation between the SCT and BTT is to be expected as a higher QoS and a lower (i.e. closer to 0) mean x-coordinate indicate better performance.

Abbreviations: BTT, baking tray task; CBS, Catherine Bergego scale; CVDT, computerized visual detection task; EWB, endpoint weightings bias; MLBT-d, McIntosh line bisection task-digitized; QoS, quality of search; SCT, star cancellation task; SLBT, Schenkenberg line bisection task; SNQ, subjective neglect questionnaire.

Supplementary Table 6 Final models of fixed-effect predictors for predicting performance in the ipsilesional side of the star cancellation task and the ipsilesional condition of the computerized visual detection task

Predictor	β[1]	$SE_β$	95% CI lower bound	95% CI higher bound	*P* value
**QoS, ipsilesional side of screen (SCT) across T0 to T5 (*n* = 22)**					
Age	-0.014	0.007	-0.027	-1.18E-04	0.048
**Weighted hits, ipsilesional condition (CVDT) across T0 to T5 (*n* = 21)**					
Time	0.443	0.206	0.034	0.852	0.034
Time x Time	-0.013	0.005	-0.023	-0.002	0.016
Time x Time x Time	6.56E-05	2.72E-05	1.15E-05	1.20E-04	0.018

[1] β coefficients are shown in reference to the active group.
Abbreviations: CI, confidence interval; CVDT, computerized visual detection task; QoS, quality of search; SCT, star cancellation task.

Supplementary Table 7 SPSS syntax that is generated and used in this work for statistical analyses of primary and secondary outcomes

Quality of search, contralesional side of screen (SCT)

* We started by focusing on potential removal of higher order interactions between group and time, and higher order effects of time, and finally the covariates. Initial model:

```
MIXED Left_QoS BY Gender Group WITH Time_continuous Age Months_since_stroke
  /CRITERIA=DFMETHOD(SATTERTHWAITE) CIN(95) MXITER(100) MXSTEP(10) SCORING(1)
    SINGULAR(0.000000000001) HCONVERGE(0, ABSOLUTE) LCONVERGE(0, ABSOLUTE) PCONVERGE(0.000001,
ABSOLUTE)
  /FIXED=Gender Time_continuous Time_continuous*Time_continuous
Time_continuous*Time_continuous*Time_continuous Age Months_since_stroke
  Group Group*Time_continuous Group*Time_continuous*Time_continuous
Group*Time_continuous*Time_continuous*Time_continuous | SSTYPE(3)
  /METHOD=ML
  /PRINT=DESCRIPTIVES  SOLUTION
  /RANDOM=INTERCEPT | SUBJECT(Subject) COVTYPE(ID)
  /REPEATED=Time | SUBJECT(Subject) COVTYPE(SP_POWER) SPCOORDS(Time_continuous)
  /SAVE=FIXPRED PRED
  /EMMEANS=TABLES(OVERALL)
  /EMMEANS=TABLES(Gender) COMPARE ADJ(BONFERRONI)
  /EMMEANS=TABLES(Group) COMPARE ADJ(BONFERRONI).
```

* Final model:

```
MIXED Left_QoS BY Group WITH Time_continuous
  /CRITERIA=DFMETHOD(SATTERTHWAITE) CIN(95) MXITER(100) MXSTEP(10) SCORING(1)
    SINGULAR(0.000000000001) HCONVERGE(0, ABSOLUTE) LCONVERGE(0, ABSOLUTE) PCONVERGE(0.000001,
ABSOLUTE)
  /FIXED=Time_continuous Group Group*Time_continuous | SSTYPE(3)
  /METHOD=REML
  /PRINT=DESCRIPTIVES  SOLUTION
  /RANDOM=INTERCEPT | SUBJECT(Subject) COVTYPE(ID)
  /REPEATED=Time | SUBJECT(Subject) COVTYPE(SP_POWER) SPCOORDS(Time_continuous)
  /SAVE=FIXPRED PRED
  /EMMEANS=TABLES(OVERALL)
  /EMMEANS=TABLES(Group) COMPARE ADJ(BONFERRONI).
```

* With use of the final model, we performed supplementary *post hoc* contrasts with Bonferroni correction to probe the (significant) interaction between time and group by testing differences between groups at specific time-points. The syntax presented below is an example and shows the code to calculate contrasts at Day = 0 (baseline). In the same way, we calculated contrasts at the other five time-points of interest, using the mean number of days (across participants) since Day 0 (i.e. Day 4.35, Day 24.45, Day 45.74, Day 53.16 and Day 138.16).

```
MIXED Left_QoS BY Group WITH Time_continuous
  /CRITERIA=DFMETHOD(SATTERTHWAITE) CIN(95) MXITER(100) MXSTEP(10) SCORING(1)
    SINGULAR(0.000000000001) HCONVERGE(0, ABSOLUTE) LCONVERGE(0, ABSOLUTE) PCONVERGE(0.000001,
ABSOLUTE)
  /FIXED=Time_continuous Group Group*Time_continuous | SSTYPE(3)
  /METHOD=REML
  /PRINT=DESCRIPTIVES  SOLUTION
  /RANDOM=INTERCEPT | SUBJECT(Subject) COVTYPE(ID)
  /REPEATED=Time | SUBJECT(Subject) COVTYPE(SP_POWER) SPCOORDS(Time_continuous)
  /SAVE=FIXPRED PRED
  /EMMEANS=TABLES(OVERALL)
  /EMMEANS=TABLES(Group) COMPARE ADJ(BONFERRONI) WITH(time_continuous=0.00).
```

* We also performed contrasts with the aim of building the graph (see Fig. 3 in the manuscript). To this end, we used the model that included both linear *and* quadratic group by time interaction terms. The syntax presented below is an example and shows the code to calculate contrasts at Day = 0 (baseline) for the primary outcome measure. In the same way, we calculated contrasts at the other five time-points of interest, as well as for building the graphs of the secondary outcome measures (see Fig. 4 in the manuscript).

```
MIXED Left_QoS BY Group WITH Time_continuous
  /CRITERIA=DFMETHOD(SATTERTHWAITE) CIN(95) MXITER(100) MXSTEP(10) SCORING(1)
    SINGULAR(0.000000000001) HCONVERGE(0, ABSOLUTE) LCONVERGE(0, ABSOLUTE) PCONVERGE(0.000001,
ABSOLUTE)
  /FIXED=Time_continuous Time_continuous*Time_continuous Group Group*Time_continuous
Group*Time_continuous*Time_continuous | SSTYPE(3)
  /METHOD=REML
  /PRINT=DESCRIPTIVES  SOLUTION
```

```
  /RANDOM=INTERCEPT | SUBJECT(Subject) COVTYPE(ID)
  /REPEATED=Time | SUBJECT(Subject) COVTYPE(SP_POWER) SPCOORDS(Time_continuous)
  /SAVE=FIXPRED PRED
  /EMMEANS=TABLES(OVERALL)
  /EMMEANS=TABLES(Group) COMPARE ADJ(BONFERRONI) WITH(time_continuous=0.00).
```

Sum of weighted hits, contralesional condition (CVDT)

```
* Initial model:

MIXED CVDT_WH_left BY Group Gender WITH Time_continuous Age Months_since_stroke
  /CRITERIA=DFMETHOD(SATTERTHWAITE) CIN(95) MXITER(100) MXSTEP(10) SCORING(1)
    SINGULAR(0.000000000001) HCONVERGE(0, ABSOLUTE) LCONVERGE(0, ABSOLUTE) PCONVERGE(0.000001,
ABSOLUTE)
  /FIXED=Group Gender Time_continuous Time_continuous*Time_continuous
Time_continuous*Time_continuous*Time_continuous Age Months_since_stroke Group*Time_continuous
Group*Time_continuous*Time_continuous Group*Time_continuous*Time_continuous*Time_continuous  | SSTYPE(3)
  /METHOD=ML
  /PRINT=DESCRIPTIVES  SOLUTION
  /RANDOM=INTERCEPT | SUBJECT(Subject) COVTYPE(ID)
  /REPEATED=Time | SUBJECT(Subject) COVTYPE(SP_POWER) SPCOORDS(Time_continuous)
  /SAVE=FIXPRED PRED
  /EMMEANS=TABLES(OVERALL)
  /EMMEANS=TABLES(Group) COMPARE ADJ(BONFERRONI)
  /EMMEANS=TABLES(Gender) COMPARE ADJ(BONFERRONI).

* Final model:

MIXED CVDT_WH_left BY Group Gender WITH Time_continuous
  /CRITERIA=DFMETHOD(SATTERTHWAITE) CIN(95) MXITER(100) MXSTEP(10) SCORING(1)
    SINGULAR(0.000000000001) HCONVERGE(0, ABSOLUTE) LCONVERGE(0, ABSOLUTE) PCONVERGE(0.000001,
ABSOLUTE)
  /FIXED=Group Gender Time_continuous Group*Time_continuous | SSTYPE(3)
  /METHOD=REML
  /PRINT=DESCRIPTIVES  SOLUTION
  /RANDOM=INTERCEPT | SUBJECT(Subject) COVTYPE(ID)
  /REPEATED=Time | SUBJECT(Subject) COVTYPE(SP_POWER) SPCOORDS(Time_continuous)
  /SAVE=FIXPRED PRED
  /EMMEANS=TABLES(OVERALL)
  /EMMEANS=TABLES(Group) COMPARE ADJ(BONFERRONI)
  /EMMEANS=TABLES(Gender) COMPARE ADJ(BONFERRONI).
```

* Note that the table output gives simple effects. We therefore used ‘Transform’ -> ‘Recode into same variables’ to recode the groups, and ran the final model again, giving us the β and *p* values of the linear effect of time for the other group.

* To perform *post hoc* contrasts at the six time-points of interest, the syntax line ‘WITH(time_continuous=…)’ was added to the final model. See above for an example of the syntax for the primary outcome measure.

Sum of weighted hits, bilateral condition (CVDT)

```
* Initial model:

MIXED CVDT_WH_bilat BY Group Gender WITH Time_continuous Age Months_since_stroke
  /CRITERIA=DFMETHOD(SATTERTHWAITE) CIN(95) MXITER(100) MXSTEP(10) SCORING(1)
    SINGULAR(0.000000000001) HCONVERGE(0, ABSOLUTE) LCONVERGE(0, ABSOLUTE) PCONVERGE(0.000001,
ABSOLUTE)
  /FIXED=Group Gender Time_continuous Time_continuous*Time_continuous
Time_continuous*Time_continuous*Time_continuous Age Months_since_stroke Group*Time_continuous
Group*Time_continuous*Time_continuous Group*Time_continuous*Time_continuous*Time_continuous | SSTYPE(3)
  /METHOD=ML
  /PRINT=DESCRIPTIVES  SOLUTION
  /RANDOM=INTERCEPT | SUBJECT(Subject) COVTYPE(ID)
  /REPEATED=Time | SUBJECT(Subject) COVTYPE(SP_POWER) SPCOORDS(Time_continuous)
  /SAVE=FIXPRED PRED
  /EMMEANS=TABLES(OVERALL)
  /EMMEANS=TABLES(Group) COMPARE ADJ(BONFERRONI)
  /EMMEANS=TABLES(Gender) COMPARE ADJ(BONFERRONI).

* Final model:

MIXED CVDT_WH_bilat BY Group Gender WITH Time_continuous
```

```
  /CRITERIA=DFMETHOD(SATTERTHWAITE) CIN(95) MXITER(100) MXSTEP(10) SCORING(1)
    SINGULAR(0.000000000001) HCONVERGE(0, ABSOLUTE) LCONVERGE(0, ABSOLUTE) PCONVERGE(0.000001,
ABSOLUTE)
  /FIXED=Group Gender Time_continuous Time_continuous*Time_continuous Group*Time_continuous | SSTYPE(3)
  /METHOD=REML
  /PRINT=DESCRIPTIVES  SOLUTION
  /RANDOM=INTERCEPT | SUBJECT(Subject) COVTYPE(ID)
  /REPEATED=Time | SUBJECT(Subject) COVTYPE(SP_POWER) SPCOORDS(Time_continuous)
  /SAVE=FIXPRED PRED
  /EMMEANS=TABLES(OVERALL)
  /EMMEANS=TABLES(Group) COMPARE ADJ(BONFERRONI)
  /EMMEANS=TABLES(Gender) COMPARE ADJ(BONFERRONI).
```

* To perform *post hoc* contrasts at the six time-points of interest, the syntax line 'WITH(time_continuous=…)' was added to the final model. See above for an example of the syntax for the primary outcome measure.

Endpoint weighting bias (MLBT-d)

```
* Initial model:

MIXED EWB BY Gender Group WITH Time_continuous Age Months_since_stroke
  /CRITERIA=DFMETHOD(SATTERTHWAITE) CIN(95) MXITER(100) MXSTEP(10) SCORING(1)
    SINGULAR(0.000000000001) HCONVERGE(0, ABSOLUTE) LCONVERGE(0, ABSOLUTE) PCONVERGE(0.000001,
ABSOLUTE)
  /FIXED=Gender Time_continuous Time_continuous*Time_continuous
Time_continuous*Time_continuous*Time_continuous Age Months_since_stroke Group Group*Time_continuous
Group*Time_continuous*Time_continuous Group*Time_continuous*Time_continuous*Time_continuous  | SSTYPE(3)
  /METHOD=ML
  /PRINT=DESCRIPTIVES  SOLUTION
  /RANDOM=INTERCEPT | SUBJECT(Subject) COVTYPE(ID)
  /REPEATED=Time | SUBJECT(Subject) COVTYPE(SP_POWER) SPCOORDS(Time_continuous)
  /SAVE=FIXPRED PRED
  /EMMEANS=TABLES(OVERALL)
  /EMMEANS=TABLES(Gender) COMPARE ADJ(BONFERRONI)
  /EMMEANS=TABLES(Group) COMPARE ADJ(BONFERRONI).

* Final model:

MIXED EWB BY Group WITH Time_continuous
  /CRITERIA=DFMETHOD(SATTERTHWAITE) CIN(95) MXITER(100) MXSTEP(10) SCORING(1)
    SINGULAR(0.000000000001) HCONVERGE(0, ABSOLUTE) LCONVERGE(0, ABSOLUTE) PCONVERGE(0.000001,
ABSOLUTE)
  /FIXED=Time_continuous Time_continuous*Time_continuous | SSTYPE(3)
  /METHOD=REML
  /PRINT=DESCRIPTIVES  SOLUTION
  /RANDOM=INTERCEPT | SUBJECT(Subject) COVTYPE(ID)
  /REPEATED=Time | SUBJECT(Subject) COVTYPE(SP_POWER) SPCOORDS(Time_continuous)
  /SAVE=FIXPRED PRED
  /EMMEANS=TABLES(OVERALL).
```

Relative deviation on contralesional lines (SLBT)

```
* Initial model:

MIXED SLBT_left BY Gender Group WITH Time_continuous Age Months_since_stroke
  /CRITERIA=DFMETHOD(SATTERTHWAITE) CIN(95) MXITER(100) MXSTEP(10) SCORING(1)
    SINGULAR(0.000000000001) HCONVERGE(0, ABSOLUTE) LCONVERGE(0, ABSOLUTE) PCONVERGE(0.000001,
ABSOLUTE)
  /FIXED=Gender Time_continuous Time_continuous*Time_continuous
Time_continuous*Time_continuous*Time_continuous Age Months_since_stroke Group Group*Time_continuous
Group*Time_continuous*Time_continuous Group*Time_continuous*Time_continuous*Time_continuous | SSTYPE(3)
  /METHOD=ML
  /PRINT=DESCRIPTIVES  SOLUTION
  /RANDOM=INTERCEPT | SUBJECT(Subject) COVTYPE(ID)
  /REPEATED=Time | SUBJECT(Subject) COVTYPE(SP_POWER) SPCOORDS(Time_continuous)
  /SAVE=FIXPRED PRED
  /EMMEANS=TABLES(OVERALL)
  /EMMEANS=TABLES(Gender) COMPARE ADJ(BONFERRONI)
  /EMMEANS=TABLES(Group) COMPARE ADJ(BONFERRONI).

* Final model:
```

```
MIXED SLBT_left BY Group WITH Time_continuous
  /CRITERIA=DFMETHOD(SATTERTHWAITE) CIN(95) MXITER(100) MXSTEP(10) SCORING(1)
    SINGULAR(0.000000000001) HCONVERGE(0, ABSOLUTE) LCONVERGE(0, ABSOLUTE) PCONVERGE(0.000001,
ABSOLUTE)
  /FIXED=Time_continuous | SSTYPE(3)
  /METHOD=REML
  /PRINT=DESCRIPTIVES  SOLUTION
  /RANDOM=INTERCEPT | SUBJECT(Subject) COVTYPE(ID)
  /REPEATED=Time | SUBJECT(Subject) COVTYPE(SP_POWER) SPCOORDS(Time_continuous)
  /SAVE=FIXPRED PRED
  /EMMEANS=TABLES(OVERALL)
  /EMMEANS=TABLES(Group) COMPARE ADJ(BONFERRONI).
```

Mean x-coordinate (BTT)

```
* Initial model:

MIXED BTT_mean_X BY Gender Group WITH Time_continuous Age Months_since_stroke
  /CRITERIA=DFMETHOD(SATTERTHWAITE) CIN(95) MXITER(100) MXSTEP(10) SCORING(1)
    SINGULAR(0.000000000001) HCONVERGE(0, ABSOLUTE) LCONVERGE(0, ABSOLUTE) PCONVERGE(0.000001,
ABSOLUTE)
  /FIXED=Gender Age Months_since_stroke Time_continuous Time_continuous*Time_continuous
Time_continuous*Time_continuous*Time_continuous Group Group*Time_continuous
Group*Time_continuous*Time_continuous Group*Time_continuous*Time_continuous*Time_continuous | SSTYPE(3)
  /METHOD=ML
  /PRINT=DESCRIPTIVES  SOLUTION
  /RANDOM=INTERCEPT | SUBJECT(Subject) COVTYPE(ID)
  /SAVE=FIXPRED PRED
  /EMMEANS=TABLES(OVERALL)
  /EMMEANS=TABLES(Gender) COMPARE ADJ(BONFERRONI)
  /EMMEANS=TABLES(Group) COMPARE ADJ(BONFERRONI).

* Final model:

MIXED BTT_mean_X BY Group WITH Time_continuous
  /CRITERIA=DFMETHOD(SATTERTHWAITE) CIN(95) MXITER(100) MXSTEP(10) SCORING(1)
    SINGULAR(0.000000000001) HCONVERGE(0, ABSOLUTE) LCONVERGE(0, ABSOLUTE) PCONVERGE(0.000001,
ABSOLUTE)
  /FIXED=Group | SSTYPE(3)
  /METHOD=REML
  /PRINT=DESCRIPTIVES  SOLUTION
  /RANDOM=INTERCEPT | SUBJECT(Subject) COVTYPE(ID)
  /SAVE=FIXPRED PRED
  /EMMEANS=TABLES(OVERALL)
  /EMMEANS=TABLES(Group) COMPARE ADJ(BONFERRONI).
```

CBS

```
* Initial model:

MIXED CBS_therapist_valids5 BY Gender Group WITH Time_continuous Age Months_since_stroke
  /CRITERIA=DFMETHOD(SATTERTHWAITE) CIN(95) MXITER(100) MXSTEP(10) SCORING(1)
    SINGULAR(0.000000000001) HCONVERGE(0, ABSOLUTE) LCONVERGE(0, ABSOLUTE) PCONVERGE(0.000001,
ABSOLUTE)
  /FIXED=Gender Time_continuous Time_continuous*Time_continuous
Time_continuous*Time_continuous*Time_continuous Age Months_since_stroke Group Group*Time_continuous
Group*Time_continuous*Time_continuous Group*Time_continuous*Time_continuous*Time_continuous | SSTYPE(3)
  /METHOD=ML
  /PRINT=DESCRIPTIVES  SOLUTION
  /RANDOM=INTERCEPT | SUBJECT(Subject) COVTYPE(ID)
  /SAVE=FIXPRED PRED
  /EMMEANS=TABLES(OVERALL)
  /EMMEANS=TABLES(Gender) COMPARE ADJ(BONFERRONI)
  /EMMEANS=TABLES(Group) COMPARE ADJ(BONFERRONI).

* Final model:

MIXED CBS_therapist_valids5 BY Group WITH Time_continuous
  /CRITERIA=DFMETHOD(SATTERTHWAITE) CIN(95) MXITER(100) MXSTEP(10) SCORING(1)
```

```
    SINGULAR(0.000000000001) HCONVERGE(0, ABSOLUTE) LCONVERGE(0, ABSOLUTE) PCONVERGE(0.000001,
ABSOLUTE)
  /FIXED=Time_continuous Time_continuous*Time_continuous | SSTYPE(3)
  /METHOD=REML
  /PRINT=DESCRIPTIVES  SOLUTION
  /RANDOM=INTERCEPT | SUBJECT(Subject) COVTYPE(ID)
  /SAVE=FIXPRED PRED
  /EMMEANS=TABLES(OVERALL).
```

SNQ

```
* Initial model:

MIXED SNQ_patient BY Gender Group WITH Time_continuous Age Months_since_stroke
  /CRITERIA=DFMETHOD(SATTERTHWAITE) CIN(95) MXITER(100) MXSTEP(10) SCORING(1)
    SINGULAR(0.000000000001) HCONVERGE(0, ABSOLUTE) LCONVERGE(0, ABSOLUTE) PCONVERGE(0.000001,
ABSOLUTE)
  /FIXED=Gender Time_continuous Time_continuous*Time_continuous
Time_continuous*Time_continuous*Time_continuous Age Months_since_stroke Group Group*Time_continuous
Group*Time_continuous*Time_continuous Group*Time_continuous*Time_continuous*Time_continuous | SSTYPE(3)
  /METHOD=ML
  /PRINT=DESCRIPTIVES  SOLUTION
  /RANDOM=INTERCEPT | SUBJECT(Subject) COVTYPE(ID)
  /SAVE=FIXPRED PRED
  /EMMEANS=TABLES(OVERALL)
  /EMMEANS=TABLES(Gender) COMPARE ADJ(BONFERRONI)
  /EMMEANS=TABLES(Group) COMPARE ADJ(BONFERRONI).

* Final model:

MIXED SNQ_patient BY Group WITH Time_continuous
  /CRITERIA=DFMETHOD(SATTERTHWAITE) CIN(95) MXITER(100) MXSTEP(10) SCORING(1)
    SINGULAR(0.000000000001) HCONVERGE(0, ABSOLUTE) LCONVERGE(0, ABSOLUTE) PCONVERGE(0.000001,
ABSOLUTE)
  /FIXED=Time_continuous Time_continuous*Time_continuous | SSTYPE(3)
  /METHOD=REML
  /PRINT=DESCRIPTIVES  SOLUTION
  /RANDOM=INTERCEPT | SUBJECT(Subject) COVTYPE(ID)
  /SAVE=FIXPRED PRED
  /EMMEANS=TABLES(OVERALL)
  /EMMEANS=TABLES(Group) COMPARE ADJ(BONFERRONI).
```

References Supplementary Material

1. Gauthier L, Dehaut F, Joanette Y. The bells test: A quantitative and qualitative test for visual neglect. *International Journal of Clinical Neuropsychology*. 1989;11(2):49-54.
2. Edgeworth JA, MacMillan TM, Robertson IH. *The Balloons Test Manual*. Thames Valley Test Company; 1998.
3. Schenkenberg T, Bradford D, Ajax E. Line bisection and unilateral visual neglect in patients with neurologic impairment. *Neurology*. 1980;30(5):509-517.
4. McIntosh RD, Ietswaart M, Milner AD. Weight and see: Line bisection in neglect reliably measures the allocation of attention, but not the perception of length. *Neuropsychologia*. 2017;106:146-158.
5. McIntosh RD, Schindler I, Birchall D, Milner AD. Weights and measures: A new look at bisection behaviour in neglect. *Cognitive Brain Research*. 2005;25(3):833-850.
6. Middag-van Spanje M, Schuhmann T, Nijboer T, Van der Werf O, Sack AT, Van Heugten C. Study protocol of transcranial electrical stimulation at alpha frequency applied during rehabilitation: A randomized controlled trial in chronic stroke patients with visuospatial neglect. *BMC Neurol*. 2022;22(1).
7. Dalmaijer ES, Van der Stigchel S, Nijboer TCW, Cornelissen THW, Husain M. CancellationTools: All-in-one software for administration and analysis of cancellation tasks. *Behav Res Methods*. 2014;47(4):1065-1075.
8. Schuhmann T, Duecker F, Middag-van Spanje M, et al. Transcranial alternating brain stimulation at alpha frequency reduces hemispatial neglect symptoms in stroke patients. *International Journal of Clinical and Health Psychology*. 2022;22(3).
